# An Airway Network Flow Assignment Approach Based on an Efficient Multiobjective Optimization Framework

**DOI:** 10.1155/2015/302615

**Published:** 2015-06-09

**Authors:** Xiangmin Guan, Xuejun Zhang, Yanbo Zhu, Dengfeng Sun, Jiaxing Lei

**Affiliations:** ^1^School of Electronic and Information Engineering, Beihang University, Beijing 100191, China; ^2^National Key Laboratory of CNS/ATM, Beijing 100191, China; ^3^Beijing Key Laboratory for Cooperative Vehicle Infrastructure Systems and Safety Control, Beijing 100191, China; ^4^School of Aeronautics and Astronautics, Purdue University, West Lafayette, IN 47907-2023, USA

## Abstract

Considering reducing the airspace congestion and the flight delay simultaneously, this paper formulates the airway network flow assignment (ANFA) problem as a multiobjective optimization model and presents a new multiobjective optimization framework to solve it. Firstly, an effective multi-island parallel evolution algorithm with multiple evolution populations is employed to improve the optimization capability. Secondly, the nondominated sorting genetic algorithm II is applied for each population. In addition, a cooperative coevolution algorithm is adapted to divide the ANFA problem into several low-dimensional biobjective optimization problems which are easier to deal with. Finally, in order to maintain the diversity of solutions and to avoid prematurity, a dynamic adjustment operator based on solution congestion degree is specifically designed for the ANFA problem. Simulation results using the real traffic data from China air route network and daily flight plans demonstrate that the proposed approach can improve the solution quality effectively, showing superiority to the existing approaches such as the multiobjective genetic algorithm, the well-known multiobjective evolutionary algorithm based on decomposition, and a cooperative coevolution multiobjective algorithm as well as other parallel evolution algorithms with different migration topology.

## 1. Introduction

With the development of civil aviation, the permanently increasing air traffic and the limited airspace resource have resulted in more and more serious congestion and flight delay [1–3]. Meantime, heavy congestion challenges the airspace safety and the flight delay costs the airline industry billions of dollars every year [4]. Hence, how to safely accommodate high levels of demand and to maximize the use of capacity-limited airspace and airport resources has become a major concern to both researchers and practitioners in air traffic management (ATM).

In recent years, FAA proposed the 4D-trajectory (4DT) operation concept in next generation air transportation system (NextGen), which includes three dimensions of position and a time description of the flight. Under the 4DT environment, flights can be accurately planned in both space and time. The airway network flow assignment (ANFA) approach can provide solutions from a global point of view with the aim of alleviating airspace congestion and reducing time cost by optimizing the 4D trajectories of all flights considered in the entire airspace. Therefore, it has become a focus of research interests.

However, the ANFA problem is a large-scale combinatorial optimization problem with complicated constraints as well as tightly coupled variables, which is difficult to solve in general. For instance, there are more than ten thousand flights flying over China every day on the air route network with more than one thousand waypoints, which generates a large number of tightly coupled decision variables and constraints. Hence, in order to get optimal flight plans for all flights, the ANFA problem has a very high computational complex. Besides, with consideration of reducing congestion and minimizing the induced delay, the problem usually has multiple objectives which are all nondifferentiable or even noncontinuous.

Due to the importance of the ANFA problem, it has drawn a mass of attention of researchers [5]. Earlier work has focused on the slot-time allocation, known as ground holding in a single or multiple airport setting, in which delays propagate through the network [6, 7]. Besides, the flight level allocation has been considered to the flights in this problem [8, 9]. In addition, Bertsimas et al. [10, 11] considered the time slots and routes assignment simultaneously through an efficient deterministic approach. From a stochastic optimization point of view, Delahaye and Odoni proposed a genetic algorithm to solve the problem [12]. However, it often falls into the local optimum because of the large-scale decision variables. More recently, a cooperative coevolution (CC) algorithm was introduced into the resolution of the ANFA problem [13]. It adopted the divide-and-conquer strategy to divide the complex problem into several low-dimensional subproblems which becomes easier to deal with.

So far these works took the minimization of the airspace congestion or the flight delay as the sole objective [14, 15]. However, it might be more appropriate to consider both airspace congestion and extra flight cost and try to seek a good trade-off between them. Hence, Daniel et al. [16] formulated the problem into a biobjective optimization problem and solved it with the multiobjective genetic algorithm (MOGA). However, the application of existing multiobjective evolutionary algorithms (MOEAs) to the ANFA problem might lead to poor performance due to the scale of the problem. To the best of our knowledge, few works could effectively solve complicated multiobjective optimization problems with thousands of variables.

With the consideration of the reduction of the airspace congestion and the flight delay simultaneously, this paper formulates the ANFA problem as a multiobjective optimization model. Then, an efficient multiobjective optimization framework is presented to solve it. This framework employs the parallel computation and population diversity adjustment techniques to improve the optimization capability. Firstly, an effective multi-island parallel evolution algorithm (PEA) with multiple evolution populations is adopted. Besides, one-way ring migration topology is applied to exchange individuals among populations to improve the efficiency of the cooperation of populations. Secondly, the multiobjective evolutionary algorithm NSGA2 is used to optimize each population. In addition, a cooperative coevolution algorithm is adapted to divide the ANFA problem into several low-dimensional biobjective optimization problems which are easier to deal with. Finally, in order to maintain the diversity of solutions and avoid prematurity, a dynamic adjustment operator based on solution congestion degree is specifically designed. It can greatly improve the distribution of the nondominated solutions in the archive and maintain the population diversity by inheriting the nondominated solutions with low congestion degree in a high probability. Simulation results using the real traffic data from China air route network and daily flight plans demonstrate that the proposed approach can improve the solution quality effectively, showing superiority to the existing approaches such as the multiobjective genetic algorithm, the well-known multiobjective evolutionary algorithm based on decomposition (MOEA/D), and a CC-based multiobjective algorithm as well as other parallel evolution algorithms with different migration topology.

The rest of this paper is organized as follows.  Section 2 introduces the formulation of the investigated biobjective ANFA problem.  Section 3 describes the proposed multiobjective optimization framework in detail. Experimental study is presented in Section 4 to evaluate the effectiveness of our algorithm. Finally, Section 5 concludes this paper and discusses directions for further research.

## 2. Problem Formulation

The airway network can be modeled as a directed graph including the waypoints as nodes and segments as edges. Each flight can be considered as a single commodity on the network with a defined pair of origin-destination nodes. Besides, each flight has its predefined departure time slots and the optional routes. In this paper, with the consideration of safety and efficiency, the ANFA problem is formulated as a biobjective problem to reduce the airspace congestion and the total flight delay simultaneously.

Each flight (flight *k*) is associated with a pair of decision variables (*r*_*k*_, *τ*_*k*_), where *r*_*k*_ is a possible route and *τ*_*k*_ is a feasible departure time slot. In addition, they are subject to the following:

1. *r*_*k*_ ∈ Path_*k*_ = {*r*_1_′, *r*_2_′,…, *r*_max_′}, where Pathk is the set of all possible paths of flight *k*;
2. *t*_*k*_^min^ ≤ *τ*_*k*_ ≤ *t*_*k*_^max^, where *t*_*k*_^max^ is the latest departure time slot and *t*_*k*_^min^ is the earliest departure time slot.

We can see that constraint (1) denotes a feasible route of each flight from a defined route set, while constraint (2) enforces the flight to depart at a predefined departure time slot.

The first objective function is the minimization of the airspace congestion. Here the workload of the sectors is used to indicate the airspace congestion, which is defined as follows.

It is generally known that the workload of a sector mainly depends on the monitoring workload and the coordination workload. Hence, the total workload of a sector *S*_*k*_ at time *t* can be roughly expressed by the following [16]:

(1) $$\begin{array}{c} W_{S_{k}}^{t}=W_{moS_{k}}^{t}+W_{coS_{k}}^{t}, \end{array}$$

where *W*_mo*S*_*k*__^*t*^ is the monitoring workload and can be numerically estimated by

(2) $$\begin{array}{c} W_{mos_{k}}=\left\{\begin{array}{cc} 1+M_{S_{k}}^{t}-C_{mS_{k}}^{t}, & if\;M_{S_{k}}^{t}>C_{mS_{k}}^{t}\\ 0, & else, \end{array}\right. \end{array}$$

where *M*_*S*_*k*__^*t*^ is related to the number of aircraft in sector *S*_*k*_ at time *t* and *C*_*mS*_*k*__^*t*^is the monitoring critical capacity of the sector at time *t*.

Besides, *W*_co*S*_*k*__^*t*^ is the coordination workload and can be numerically estimated by

(3) $$\begin{array}{c} W_{cos_{k}}=\left\{\begin{array}{cc} 1+C_{S_{k}}^{t}-C_{cS_{k}}^{t}, & if\;C_{S_{k}}^{t}>C_{cS_{k}}^{t}\\ 0, & else, \end{array}\right. \end{array}$$

where *C*_*S*_*k*__^*t*^ is related to the flights passing through the boundary of sector *S*_*k*_ at time *t* and *C*_*S*_*k*__^*t*^ is related to the number of aircraft passing the boundaries of sector *S*_*k*_ at time *t*.

The goal of the optimization is to minimize the airspace congestion via spreading the congestion over several sectors. Hence, the objective is defined by the following [16]:

(4) $$\begin{array}{c} minAC=\overset{k=n_{S}}{\underset{k=1}{\sum }}\left({\left(\underset{t\in T}{\sum }W_{S_{k}}^{t}\right)}^{1-\varphi }\times {\left(\underset{t\in T}{max}W_{S_{k}}^{t}\right)}^{\varphi }\right), \end{array}$$

where *φ*, (1 − *φ*)∈[0,1] indicates the relative importance of the maximum congestion and the average congestion.

The second objective is the minimization of the extra flight cost (EFC) which includes the departure delay and the airborne delay caused by choosing a longer path than the shortest one. Then the second objective can be expressed as follows [16]:

(5) $$\begin{array}{c} \min EFC=\overset{}{\underset{i\in F}{\sum }}{\left(\left|\tau_{i}-t_{k}^{prf}\right|+\left(t_{r_{i}}-t_{r_{i}^{prf}}\right)\right)}^{2}, \end{array}$$

where *t*_*r*_*i*__ and *t*_*r*_*i*_^prf^_ denote the flight time of *r*_*i*_ and the preferred shortest path, respectively, and *t*_*k*_^prf^ indicates the preferred departure time. The first part is the airborne delay, and the second part is the ground delay.

## 3. Optimization Method

It can be seen that the ANFA problem is a large-scale combinatorial optimization problem, and the objective functions are nonlinear and nondifferentiable. Black-box optimization methods such as EAs appeared to be promising to deal with this kind of problem [15–19]. Given the multiobjective model of the ANFA problem, basically any existing MOEA for discrete multiobjective optimization can be readily utilized. However, the ANFA problem involves thousands of tightly coupled decision variables which are difficult to solve. In addition, few existing MOEAs have been extensively investigated on problems of such a large scale while the scalability of EAs with respect to the number of decision variables is in general deemed to be poor; therefore a direct application of existing MOEAs might not obtain satisfactory solutions.

With the aim of avoiding prematurity and improving the convergence rate of this complex problem, a new multiobjective optimization framework is proposed. Firstly, an effective multi-island parallel evolution algorithm with multiple evolution populations is employed and one-way ring migration topology for exchange individuals among populations is applied to improve the efficiency of the cooperation of populations. Secondly, the multiobjective evolutionary algorithm NSGA2 is used to optimize each population. In addition, this paper introduces the idea of cooperative coevolutionary (CC) into the resolution of the multiobjective ANFA problems. The main idea is to divide the high-dimensional problem into low-dimensional subcomponents. The subcomponents work cooperatively to obtain better solutions. Finally, all the nondominated solutions are stored in an archive. As the ANFA problem is of such a large scale with thousands of variables, the main difficulty is how to maintain the diversity of the population to get better solutions and to improve the distribution of the solutions in the archive. Hence, in order to maintain the diversity of solutions and to avoid prematurity, a dynamic adjustment operator based on solution congestion degree is specifically designed for the ANFA problem. The framework is presented in Algorithm 1.

### 3.1. Multi-Island Parallel Evolution Algorithm

As parallel computers become more commonplace in scientific computing, it becomes more feasible to harness their power for use with evolutionary algorithms (EAs) [20]. Multi-island PEAs consist of several populations, which can optimize simultaneously to avoid premature convergence. They have been successfully applied to find acceptable solutions to problems in different engineering domains [21, 22].

Suppose that there are *M* islands and *N* flights. Then each population can be denoted as

(6) $$\begin{array}{c} pop_{i}=\left\{idiv_{i1},idiv_{i2},\ldots ,idiv_{ips}\right\},\;1\leq i\leq M, \end{array}$$

where *idiv*_*ij*_ is defined by

(7) $$\begin{array}{c} idiv_{ij}=\left(r_{ij1},\tau_{ij1},r_{ij2},\tau_{ij2},\ldots ,r_{ijN},\tau_{ijN}\right),\;1\leq j\leq ps, \end{array}$$

where ps is the size of population.

### 3.2. Migration Topology

The migration topology is a key feature of the island model which determines the destination of the migrants, and it could greatly affect the quality of the solutions and the efficiency of algorithms. If two populations rarely communicate with each other, it is difficult for the best solution to spread which may prevent populations finding better solutions.

Currently, the main migration topologies are the one-way ring topology and the random topology [22]. The random topology delivers the migrants to a randomly selected population. However, it may result in inefficient communication. After many times of migration under the random topology, the best solution cannot be spread effectively among populations. In the one-way ring topology, populations are numbered, and the worst individual of a population is replaced by the best individual of the next population. The one-way ring topology can provide sufficient communication among populations and maintain the population diversity, which can effectively avoid premature convergence and improve the optimization capability.

### 3.3. Cooperative Coevolution for Each Population

Though the multi-island PEA uses several populations simultaneously, in fact each population is hard to avoid falling into a local optimum because the ANFA problem involves large-scale tightly coupled decision variables as presented in the previous sections. Hence, we introduce the cooperative coevolution algorithm for the optimization of each population to further improve the solution quality. The cooperative coevolution (CC) algorithm, adopting the divide-and-conquer strategy, divides the complex problem into several low-dimensional subproblems [23–25]. There are two critical issues in this approach.

*(1) Decomposition Strategy*. The decomposition strategy is a key feature of the cooperative coevolution framework which can greatly affect the capability and the efficiency of algorithms. In this work, the random grouping strategy is used which has been both theoretically and experimentally proved to be effective for the large-scale complex problem [23]. At each generation, each population is randomly divided into *ns* disjoint subpopulations with the same population size:

(8) popi=spi1,…,spins, 1≤i≤Mspij=sidij1,sidij2,…,sidijps, 1≤j≤nssidijk=rjk1′,τjk1′,rjk2′,τjk2′,…,rjkN/ns′,τjkN/ns′,000000000000000000000000000001≤j≤ps,

where *sid* denotes the individual of each subpopulation and sp_*i*_^*j*^ indicates the subpopulation *j* of population *i*.

*(2) Subpopulation Optimization*. Another critical point is optimization for each subpopulation. In this paper, the well-known multiobjective evolutionary algorithm NSGA2 is employed by each subpopulation [26]. Besides, differential evolution (DE) [27] is used in the framework to generate new solutions, because it is a simple yet effective algorithm for global optimization. DE is a randomized parallel searching algorithm. It begins with a random population, according to specific rules, for example, selection, crossover, and mutation. An optimized resolution is reached by retaining good individuals and discarding bad individuals. Compared with other optimization algorithms, DE has the advantages in global optimization as well as easy operation. Its operators are described below.

Suppose that *f*(*x*) is the objective function and the goal is to minimize it.

In mutation, if current chromosome is *x*_*i*,*G*_, then choose three different chromosomes from current generation population named *x*_*r*1,*G*_, *x*_*r*2,*G*_, and *x*_*r*3,*G*_. The mutation operator is defined by

(9) $$\begin{array}{c} V_{i,G+1}=x_{r1,G}+F\cdot \left(x_{r2,G}-x_{r3,G}\right),\;F\in \left[0,2\right], \end{array}$$

where *F* is a parameter which decides the scale of mutation. A new chromosome is generated by the crossover of *V*_*i*,*G*+1_ and *x*_*i*,*G*_ as follows:

(10) $$\begin{array}{c} U_{i,G+1}=\left(u_{1i,G+1},u_{2i,G+1},\ldots ,u_{Ni,G+1}\right)\\ u_{j,i,G+1}=\left\{\begin{array}{cc} v_{ji,G+1}, & rand\left(j\right)\leq CR\vee j=rnbr\left(i\right)\\ x_{ji,G}, & else, \end{array}\right. \end{array}$$

where rnbr(*i*) is a random integer number between 1 and *N*, which ensures that *U*_*i*,*G*+1_ gets at least one component from *v*_*i*,*G*+1_ component from *V*_*i*,*G*+1_ and rand(*j*) is a uniformly distributed random number between 0 and 1.

After the evaluation of each chromosome, the chromosomes of next generation are chosen according to the following rule:

(11) $$\begin{array}{c} x_{i,G+1}=\left\{\begin{array}{cc} u_{i,G+1}, & f\left(u_{i,G+1}\right)\leq f\left(x_{i,G}\right)\\ x_{i,G}, & else. \end{array}\right. \end{array}$$

### 3.4. Dynamic Adjustment Operator Based on Solution Congestion Degree

As the optimization generation increases, the population diversity decreases rapidly for more solutions congested at a local searching space [28, 29]. On the one hand, too crowded solutions will cause premature convergence. On the other hand, some local searching space with sparse solutions is not explored enough and needs more attention for better solutions. Hence, the more evenly the nondominated solutions distribute, the better the optimization is. In this paper, we propose a dynamic adjustment operator to improve the distribution of solutions and maintain the population diversity based on the congestion degree of solutions.

*(1) Congestion Degree of the Nondominated Solutions in the Archive*. The distance between solution *i* and *j* in archive is defined by

(12) $$\begin{array}{c} d_{i,j}=\frac{1}{2}\sqrt{{\left(P_{i}^{fit\left[1\right]}-P_{j}^{fit\left[1\right]}\right)}^{2}+{\left(P_{i}^{fit\left[2\right]}-P_{j}^{fit\left[2\right]}\right)}^{2}}, \end{array}$$

where *p*_*i*_^fit[1]^ and *p*_*i*_^fit[2]^ denote the value of the first and the second objective functions of solution *i*, respectively. Besides, the average distance of all solution pairs in the archive is described by

(13) $$\begin{array}{c} s=\frac{2}{n\left(n-1\right)}\overset{i=n}{\underset{i=1\;}{\sum }}\overset{j=n}{\underset{\;j=i+1}{\sum }}d_{i,j}, \end{array}$$

where *n* is the number of the solutions in archive.

Then the congestion degree of a nondominated solution can be expressed as

(14) $$\begin{array}{c} d_{i}=\frac{1}{n-1}\overset{j=n}{\underset{j=1,j\neq i}{\sum }}\delta_{i,j}, \end{array}$$

where

(15) $$\begin{array}{c} \delta_{i,j}=\left\{\begin{array}{cc} 1 & if\;d_{i,j}\leq \gamma \cdot s\\ 0 & if\;d_{i,j}>\gamma \cdot s, \end{array}\right. \end{array}$$

where *γ* is a parameter which can be predefined. We can see that the larger *d*_*i*_ is, the closer to other solutions solution *i* is.

*(2) Update the Archive*. The solutions in the archive will be constantly updated during the evolution of the subpopulations. When the number of the nondominated solutions exceeds the maximum size of the archive, the solutions *i* will be moved out from the archive in a probability

(16) $$\begin{array}{c} p_{i}=\frac{{d_{i}}^{\alpha }}{\sum_{j=1}^{n}{d_{j}}^{\alpha }}, \end{array}$$

where *α* is a positive regulation factor. Equation (16) indicates that the higher *d*_*i*_ is, the higher probability it will be moved out from the archive.

## 4. Experimental Studies

### 4.1. Database and Experimental Setup

**Figure 1:**
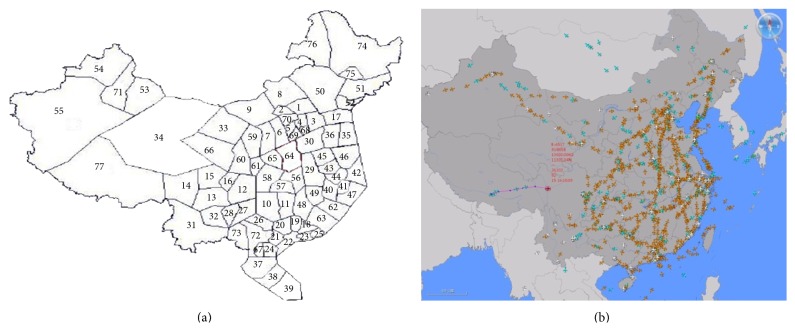
(a) Airspace sectors in China. (b) Flights operation in China.

The national route network of China consists of 1706 airway segments, 940 waypoints, and 150 airports. Note that the takeoff and landing phases of flights are truncated within a given radius (usually 10 NM) around airports. The traffic around airports is managed with specific procedures by the terminal control area (TCA) control services in these zones. The airspace is divided into many sectors, and Figure 1(a) shows the sectored airspace in China. The air traffic data was extracted from flight schedule database (FSD) of the summer in 2009 released by Civil Aviation Administration of China (CAAC). In order to better describe the difference between the algorithms' performances, we consider two scenarios: 960 flights (the busiest one hour) and 1664 flights (the busiest three hours).

The parameters are set as follows: the number of populations *M* = 5, *ϕ* = 0.9, *φ* = 0.1, *r* = 0.3, *γ* = 1/25, and *ns* = 10. The mutation probability and the crossover probability of DE are 0.15 and 0.85.

The algorithms, such as our proposed method, MOGA, MOEA/D [30], and cooperative coevolution based algorithm, in this work were implemented in C++, and the simulations were performed on a server with an E5620 2.4 GHz CPU with 12 GB RAM. For each algorithm, the results were collected and analyzed on the basis of 15 independent runs. Besides, the proposed approach was realized by multithreaded programming. Then, the optimization of all islands and all subcomponents of each population can proceed separately and simultaneously which can reduce the computation time.

The parameters used in all experiments are listed in Table 1.

In order to evaluate the performance of the solutions obtained by each of the algorithms, three typical metrics are adopted: the convergence metric (*γ*) [26], the spread metric (Δ) [31], and the hypervolume metric *I*_*H*_ [32, 33]. *γ* suggests the average Euclidean distance from the obtained nondominated solution set to the actual Pareto front. Note that it is difficult to find the actual Pareto front for most real-world optimization problems; so we use the best solution set obtained by these algorithms in 15 runs. Δ indicates the diversity of solutions along the Pareto front. *I*_*H*_ can evaluate the convergence and the extent of spread of the solutions simultaneously without the real Pareto front. The smaller the first two indexes are, the better the algorithm is. On the contrary, the larger the third index is, the better the algorithm is.

### 4.2. Comparison with the Existing Methods

In order to test the effectiveness of the proposed multi-island PEA framework, in this part, we will compare it with some existing algorithms, including the classical multiobjective genetic algorithm (MOGA), multiobjective evolutionary algorithm based on decomposition (MOEA/D), and a CC-based multiobjective algorithm (CCMA).

Tables 2 and 3 show the average value of *I*_*H*_, *I*_*D*_, and Δ over 15 independent runs of the algorithms for the two scenarios, respectively. In each row of the table, the best value is highlighted in boldface. It can be seen from the tables that PEA outperforms the other three algorithms in terms of *I*_*H*_, *I*_*D*_, and Δ. Moreover, when the number of flights increases, PEA performs much better. It can be concluded that PEA has superiority to solve this large-scale problem.

**Figure 2:**
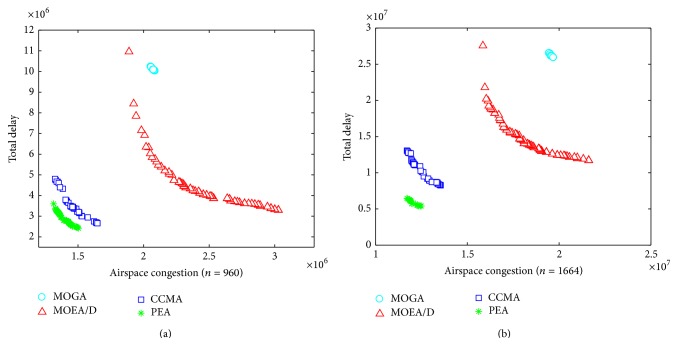
Comparison of different algorithms for 960 flights (a) and 1664 flights (b).

Figure 2 shows the nondominated solutions obtained by the four algorithms. Specifically, the nondominated solutions of each algorithm were obtained over 15 runs. From Figure 2, it can be concluded that PEA performs the best because its solutions can dominate those obtained by other algorithms. Besides, it can be seen that MOGA has the worst performance, and CCMA performs better than MOEA/D, but MOEA/D has good performance in terms of diversity.

From the experimental results, we find that PEA performs better than the other three methods for the two scenarios. It adopts an effective multi-island parallel evolution framework which can improve the optimization capability. Besides, the one-way ring migration strategy can further avoid premature. In addition, this paper introduces the cooperative coevolutionary (CC) into each population optimization via dividing the high-dimensional problem into low-dimensional subcomponents. The subcomponents work cooperatively to obtain better solutions. With the help of parallel computation, the computation is just about 30 minutes which is feasible for the ANFA problem.

### 4.3. Investigation of the Effectiveness of the Dynamic Adjustment Operator

In the previous section, the first set of experiments has justified the superiority of PEA to existing methods. The next experiment is designed to further investigate whether the dynamic adjustment operator (DAO) based on solution congestion degree contributes to the success of PEA.

Tables 4 and 5 show the results obtained by PEA and PEA without DAO in terms of the values of the metrics over 15 independent runs of the algorithms when the number of flights is 996 and 1664, respectively. It is seen from the tables that PEA always outperforms the other algorithm in terms of *I*_*H*_, *I*_*D*_, and Δ.

**Figure 3:**
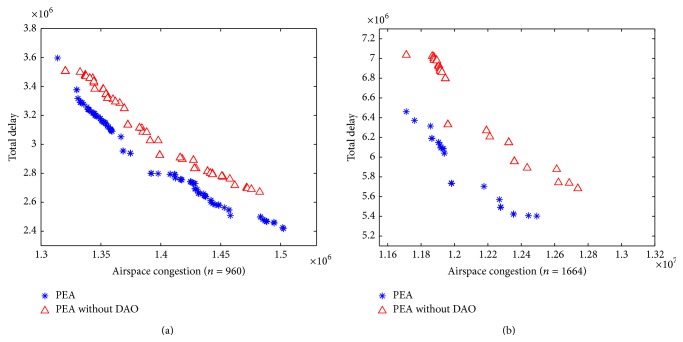
Comparison of PEA and PEA without DAO for 960 flights (a) and 1664 flights (b).

Furthermore, like the first experiment, the nondominated solutions of the algorithms are shown in Figure 3. It shows that PEA performs much better than the other method and its nondominated solutions can dominate the solutions obtained by PEA without DAO. For the scenario of 1664 flights, PEA has the most nondominated solutions and spreads nicely in the objective space.

As the optimization generation increases, more solutions will be congested in some local searching space which may cause premature convergence. The dynamic adjustment operator based on the congestion degree can effectively improve the distribution of solutions by inheriting the nondominated solutions with low congestion degree in a high probability. Hence, it can avoid decreasing the diversity of all populations.

## 5. Conclusion and Future Work

With the aim of reducing the airspace congestion and the flights delay simultaneously, this paper formulates the airway network flow assignment (ANFA) problem into a multiobjective optimization model and presents a new multiobjective optimization framework to solve it. Firstly, an effective multi-island parallel evolution algorithm is employed to solve the problem by multiple evolution populations. Besides, one-way ring migration topology is applied to improve the efficiency of the cooperation of populations by exchanging individuals among populations. Secondly, the multiobjective evolutionary algorithm NSGA2 is used to optimize each population. In addition, a cooperative coevolution algorithm is adapted to improve the optimization capability by dividing the ANFA problem into several low-dimensional biobjective optimization problems. Finally, in order to maintain the diversity of solutions and avoid prematurity, a dynamic adjustment operator based on solution congestion degree is specifically designed. Simulation results using the real traffic data from the China air route network and daily flight plans demonstrate that the proposed approach can improve the solution quality effectively, showing superiority to the existing approaches such as the multiobjective genetic algorithm, the well-known multiobjective evolutionary algorithm based on decomposition, and a CC-based multiobjective algorithm as well as other parallel evolution algorithms with different migration topology. For future research, the ANFA problem with the influence of severe weather will be considered.

## Figures and Tables

**Algorithm 1:**
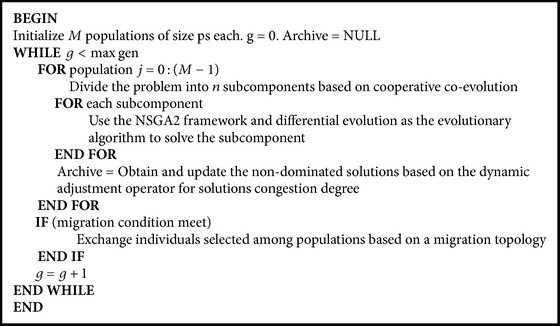
The framework of the proposed algorithm.

**Table 1:** Parameters of the experiments.

Parameters	Description	MOGA	MOEA/D	CCMA	PEA
ps	Population size	100	100	100	20 ∗ 5 (IM = 5)
max⁡gen	Max generation	150	150	150	150
*p*_*c*_	Crossover probability	0.9	0.9	0.9	0.9
*p*_*m*_	Mutation probability	0.1	0.1	—	—

**Table 2:** Comparison of different algorithms for 960 flights (*I*_*H*_, *γ*, Δ).

**Algorithm**	*I*_*H*_	*γ*	Δ
MOGA	1.1156*e* + 13	6.6865*e* + 06	1.0078
MOEA/D	1.9054*e* + 13	1.8715*e* + 06	1.2554
CCMA	3.1758*e* + 13	3.3631*e* + 05	1.0540
PEA	3.2575**e** + 13	3.1237**e** + 04	**1.0047**

**Table 3:** Comparison of different algorithms for 1664 flights (*I*_*H*_, *γ*, Δ).

**Algorithm**	*I*_*H*_	*γ*	Δ
MOGA	3.3979*e* + 12	2.1876*e* + 07	1.0113
MOEA/D	7.4733*e* + 13	1.1361*e* + 07	1.0452
CCMA	1.8763*e* + 14	4.7800*e* + 06	0.9897
PEA	2.1909**e** + 14	2.6247**e** + 05	**0.8029**

**Table 4:** Comparison of different algorithms for 960 flights (*I*_*H*_, *I*_*D*_, Δ).

**Algorithm**	*I*_*H*_	*I*_*D*_	Δ
PEA without DAO	9.2026*e* + 10	7.0467*e* + 04	1.2471
PEA	1.3937**e** + 11	3.3957**e** + 04	1.0050

**Table 5:** Comparison of different algorithms for 1164 flights (*I*_*H*_, *I*_*D*_, Δ).

**Algorithm**	*I*_*H*_	*I*_*D*_	Δ
PEA without DAO	7.3890*e* + 6	3.4212*e* + 05	0.9395
PEA	1.3107**e** + 7	1.2861**e** + 05	**0.7091**
